# Supplementary material for: Reconfigurable perovskite X-ray detector for intelligent imaging
Source: Nat Commun. 2024 Feb 27;15:1769. doi: 10.1038/s41467-024-46184-0 (PMC10899650; doi:10.1038/s41467-024-46184-0)
Supplement: Supplementary file 1 — Supplementary Information [file 41467_2024_46184_MOESM1_ESM.pdf]

Reconfigurable perovskite X-ray detector for intelligent imaging

Jincong Pang[1], Haodi Wu[1], Hao Li[1], Tong Jin[1], Jiang Tang[1,2], Guangda Niu[1,2*]

1. Wuhan National Laboratory for Optoelectronics and School of Optical and Electronic Information, Huazhong University of Science and Technology, Wuhan 430074, China.

2. Optical Valley Laboratory, Wuhan 430074, China.

Email: guangda_niu@hust.edu.cn

Table of Contents

Supplementary Figures 1

Supplementary Figure 1. Energy band for each functional layer 1

Supplementary Figure 2. Characterization of C_{60} layer 2

Supplementary Figure 3. The binding energy of the Pb element 3

Supplementary Figure 4. The detailed thermally stimulated current results 4

Supplementary Figure 5. The influence of the carriers collection capabilities 4

Supplementary Figure 6. The stability of the N-I-P $CsPbBr_3$ device 5

Supplementary Figure 7. The response and decay time. 5

Supplementary Figure 8. Schematic diagram of the heterogeneous integration 6

Supplementary Figure 9. Pictures of the heterogeneous integration process 7

Supplementary Figure 10. The result of imaging in reality and simulation 8

Supplementary Figure 11. The imaging setup for edge extraction 9

Supplementary Figure 12. Sampling data for other iron sheets 10

Supplementary Figure 13. The accuracy for another calculation process 10

Supplementary Figure 14. The evolution process for another calculation process ... 11

Supplementary Figure 15. The incident X-ray fluence with the gold-target tube 11

Supplementary Tables 12

Supplementary Table 1. The parameters of products of various companies 12

Supplementary Table 2. The parameters of slip-ring products 12

Supplementary Table 3. Glow discharge mass spectrometry data 13

Supplementary Table 4. Response time comparison of X-ray detectors 14

Supplementary Table 5. Response time comparison of visible photo-detectors 15

Supplementary Table 6. Comparison chart of perovskite X-ray detector 16

Supplementary Notes 17

Supplementary Note 1. The amount of data generated by X-ray detectors 17

Supplementary Note 2. Thermally stimulated current and its fitting analysis 19

Supplementary Note 3. Simulation of X-ray detector sensitivity 21

Supplementary Note 4. Design methodology on the bias voltages 22

Supplementary Note 5. Additional notes on the linear dynamics range 22

Supplementary References 23

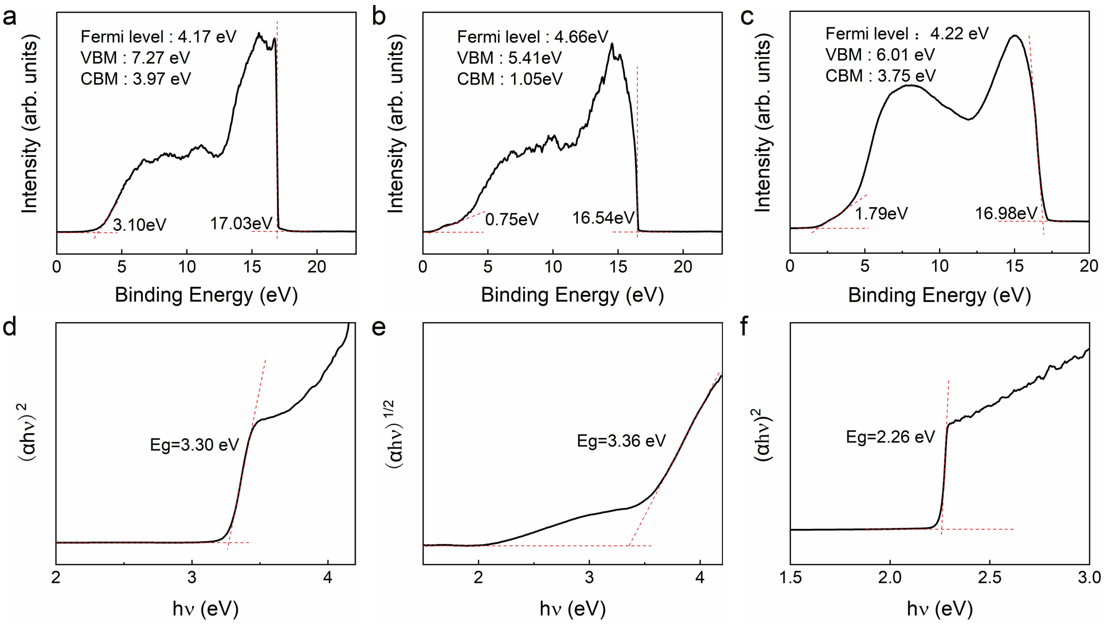

Supplementary Figure 1. Energy band for each functional layer by ultraviolet photoelectron spectroscopy and absorption spectroscopy. Figures (a, d), (b, e), and (c, f) represent ZnO, NiO_x, and C_{60}, respectively. It can be observed that magnetron sputtering ZnO was an n-type semiconductor, and NiO_x was a p-type semiconductor. The thermally evaporated C_{60} had valence and conduction bands close to those of $CsPbBr_3$, ranging from 3.3 to 5.6 eV [1], or 3.18 to 5.47 eV [2]. CBM, conduction band minimum; VBM, valence band maximum.

Source data are provided as a Source Data file.

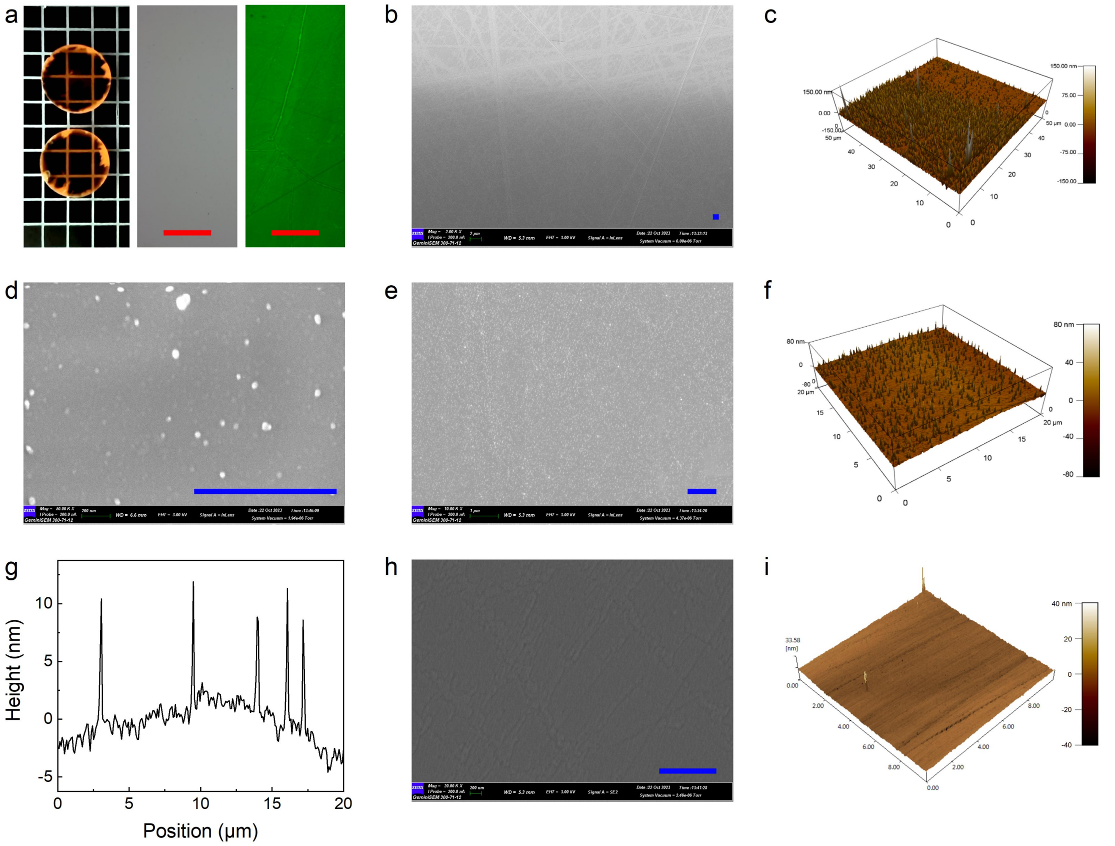

Supplementary Figure 2. Characterization of the sample with or without the C_{60} layer. (a) Sample photos taken by mobile phones, optical microscopes, and fluorescence microscopes. The scale bars in red color are 100 μm. (b-c) are figures for the areas with C_{60} only in some places and without C_{60} in others. (d-g) are figures for the areas with C_{60} all over the perovskite single crystal (PSC). Figure g shows a certain row of data in Figure f. (h-i) are figures for the areas without C_{60}. The roughness of the crystal surface in (i) is R_a = 1.15 nm, and the roughness of the C_{60} surface in (f) is R_a = 2.84 nm. The ellipsometer (UVISEL Plus, HORIBA) was used to obtain the C_{60} thickness as about 6 nm. The scale bars in blue color are 1 μm, in SEM figures b, d, e and h.

Source data are provided as a Source Data file.

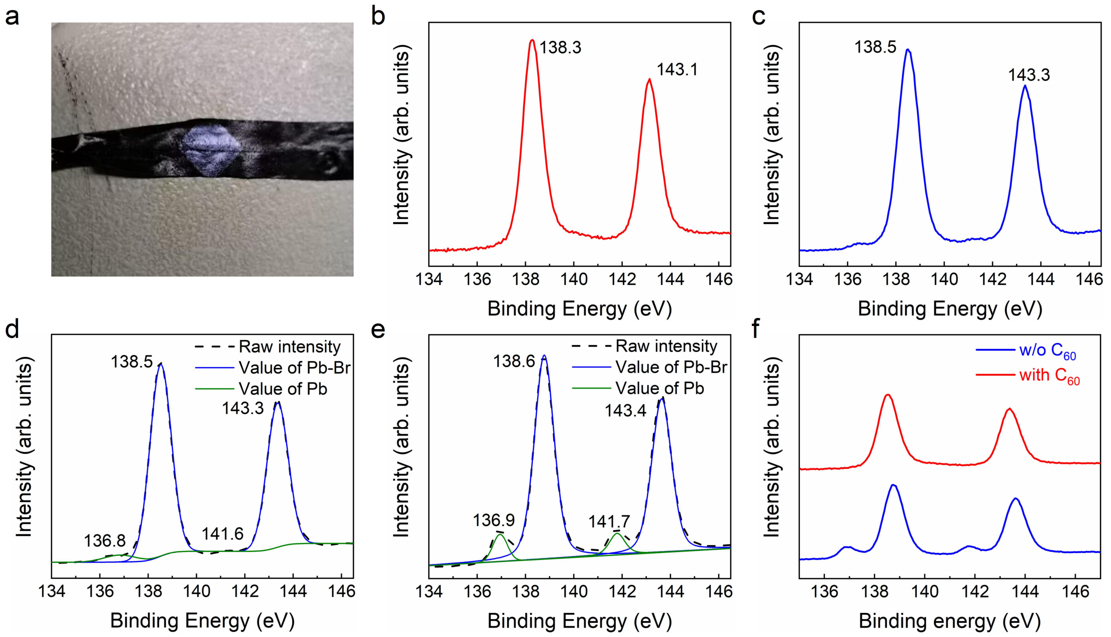

Supplementary Figure 3. The binding energy of the Pb element. Figure a shows the non-destructive removal of functional layers on the perovskite single crystal (PSC) device using adhesive tape[3]. Figure b shows the XPS result after removing the functional layer on the surface of the ZnO-C_{60}-$CsPbBr_3$ PSC device. It can be observed that the Pb element exhibits no shifted peaks with low binding energy. Figures c-d show the X-ray photoelectron spectroscopy (XPS) result after removing the functional layer on the surface of the ZnO-$CsPbBr_3$ device, with a 100 W magnetron sputtering power process. Figure e is the XPS result with a 200 W magnetron sputtering power process, which shows larger damage than that with low power sputtering. Figure f enlarges Supplementary Figure 2a. By comparing the results above, it can be observed that a few nm of C_{60} passivates the defects on the surface and protects the perovskite from being damaged by magnetron-sputtering cluster ions.

Source data are provided as a Source Data file.

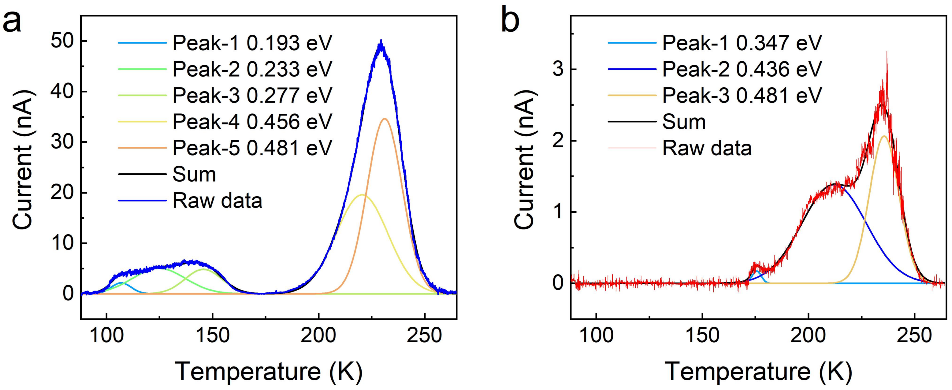

Supplementary Figure 4. The detailed thermally stimulated current results. Figures a and b show the thermally stimulated current (TSC) results of devices without and with C_{60}, respectively. The legends show the defect depth values obtained by the fitting analysis. Among them, the density of the defects with a depth of 0.193, 0.233, and 0.277 eV is 1.24×10^{14} cm^{-3}, and the density of defects with a depth of 0.456 and 0.481 eV is 5.00×10^{14} cm^{-3}. And the total defect density in Figure b is about 0.35×10^{14} cm^{-3}. Detailed method can be viewed in Supplementary Note 2.

Source data are provided as a Source Data file.

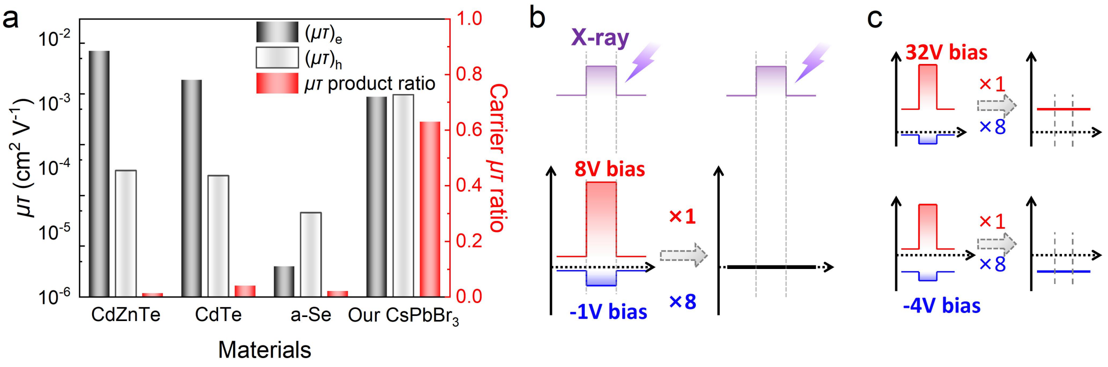

Supplementary Figure 5. The influence of the collection capabilities of the two carriers. (a) The mobility-lifetime product ($\mu\tau$) of various traditional X-ray detection materials on literature reports. The red histogram presents the ratio of the $\mu\tau$ product of the two carriers[4-6]. (b) In the ideal case, the electron and hole collection capabilities are equal. (c) In reality, the electron and hole collection capabilities are not equal. The upper figure assumes that the carrier collection capability indicated by the red color becomes one-fourth that of Figure b, and the lower figure assumes that the carrier collection capacity indicated by blue becomes one-fourth. The dark current is determined by the carrier with poor collection capability.

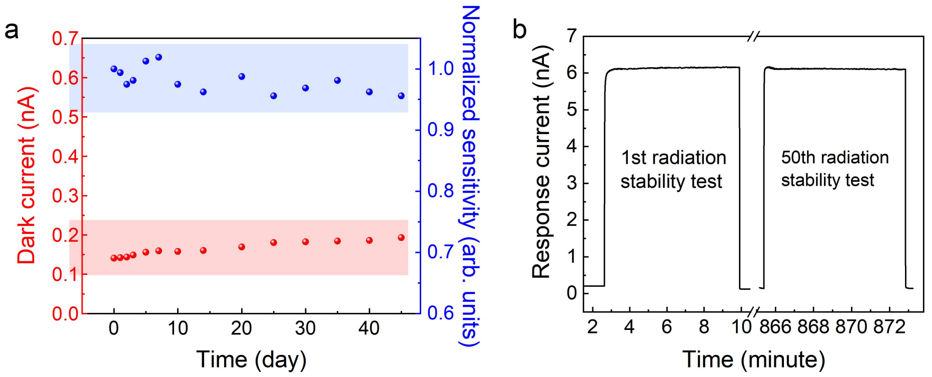

Supplementary Figure 6. The stability of the N-I-P $CsPbBr_3$ perovskite single crystal device. The perovskite single crystal (PSC) device was exposed to air and exhibited acceptable stability, with no significant decrease in performance observed after a month, as shown in Figure a. Furthermore, the device demonstrated good irradiation stability under continuous high-dose radiation, maintaining sensitivity and dark current. Figure b confirms the excellent irradiation stability of the N-I-P structure device, which was irradiated from the Bi-ZnO side. Each test was conducted at a radiation dose of approximately 1 Gy_{air}.

Source data are provided as a Source Data file.

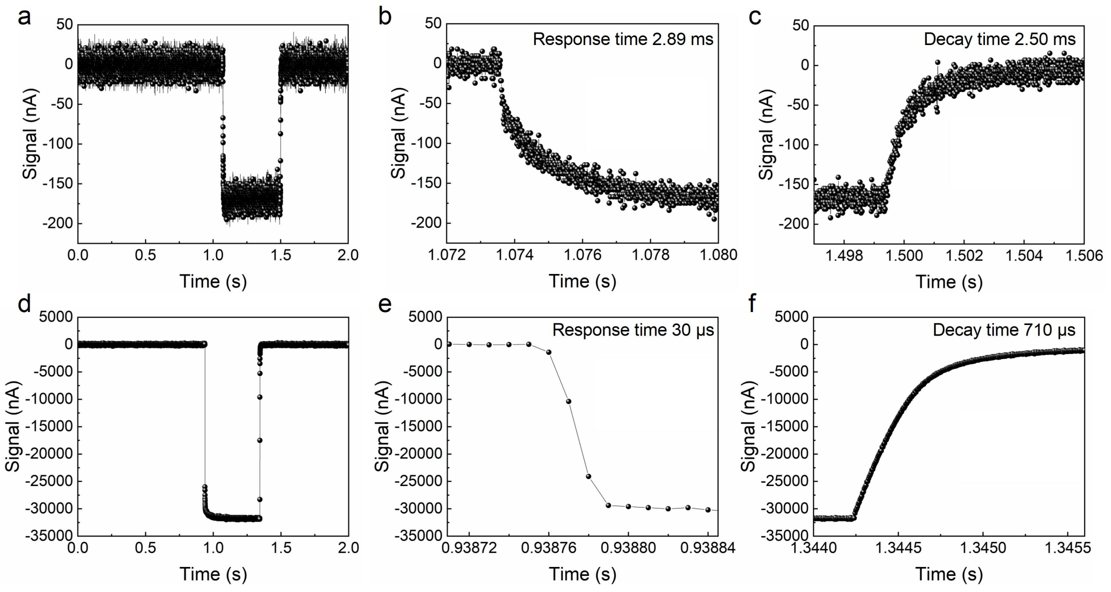

Supplementary Figure 7. The response and decay time. (a-c) Photo-response to X-ray under different abscissas. (d-f) Photo-response to X-ray. The response time is represented by the time it takes for the signal to rise from 10% to 90%, and the decay time is represented by the time it takes for the signal to fall from 90% to 10%.

Source data are provided as a Source Data file.

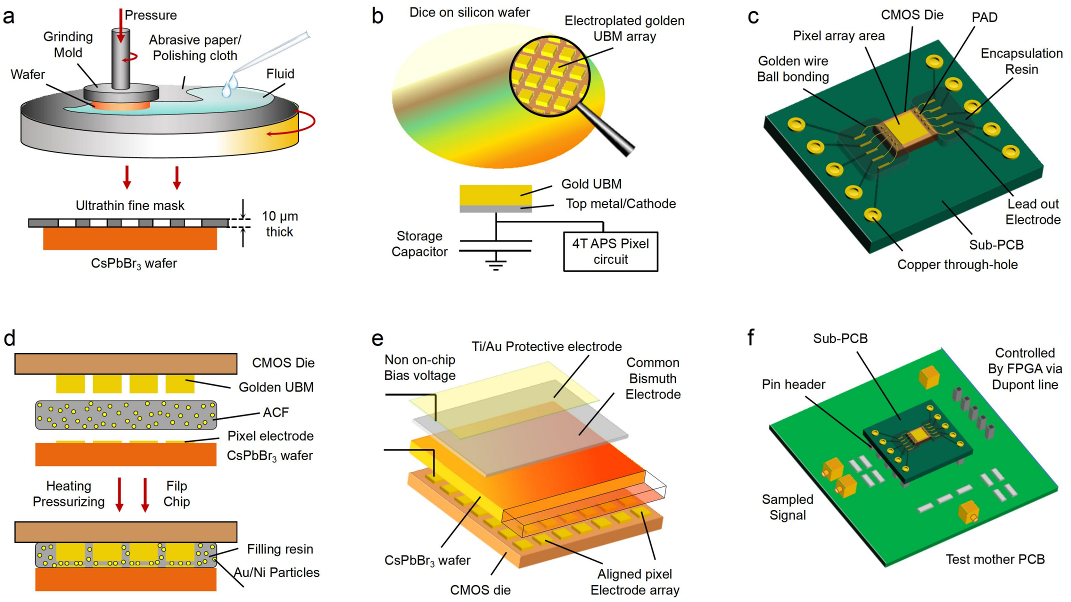

Supplementary Figure 8. Schematic diagram of the heterogeneous integration process. (a) The above figure shows the chemical mechanical polishing process of the perovskite single crystal (PSC). The figure below shows the process of vacuum method preparation of pixelated electrodes using the ultrathin fine mask. (b) After tape-out, golden under-bump metals (UBM) need to be electroplated on the silicon wafer. The figure below shows the circuit diagram of a single pixel. The design ideas can be found in our previous work[7]. APS represents active-pixel sensor. (c) Structure of sub-printed circuit board (PCB). The bottom of complementary metal-oxide-semiconductor (CMOS) die is fixed on the sub-PCB, golden wire ball bonding is used to draw out the electrical signals of the CMOS, and encapsulation resin is used to protect wires. PAD represents peripheral access device. (d) Schematic diagram of the flip-chip bonding process. First, a bilateral microscope is used to align the pixelated electrodes on the PSC and CMOS, and then anisotropic conductive tape (ACF) is added to realize the electrical connection. The UBM can enter the resin, squeeze the Au/Ni conductive particles, and conduct pixel electrodes up and down under pressure. The resin is solidified by slowly heating it while applying pressure. (e) Schematic diagram of the preparation of the common electrode and the protective electrode on the $CsPbBr_3$ PSC. (f) Mother PCB is connected to sub-PCB through pin headers. The test process is controlled by Field Programmable Gate Array (FPGA).

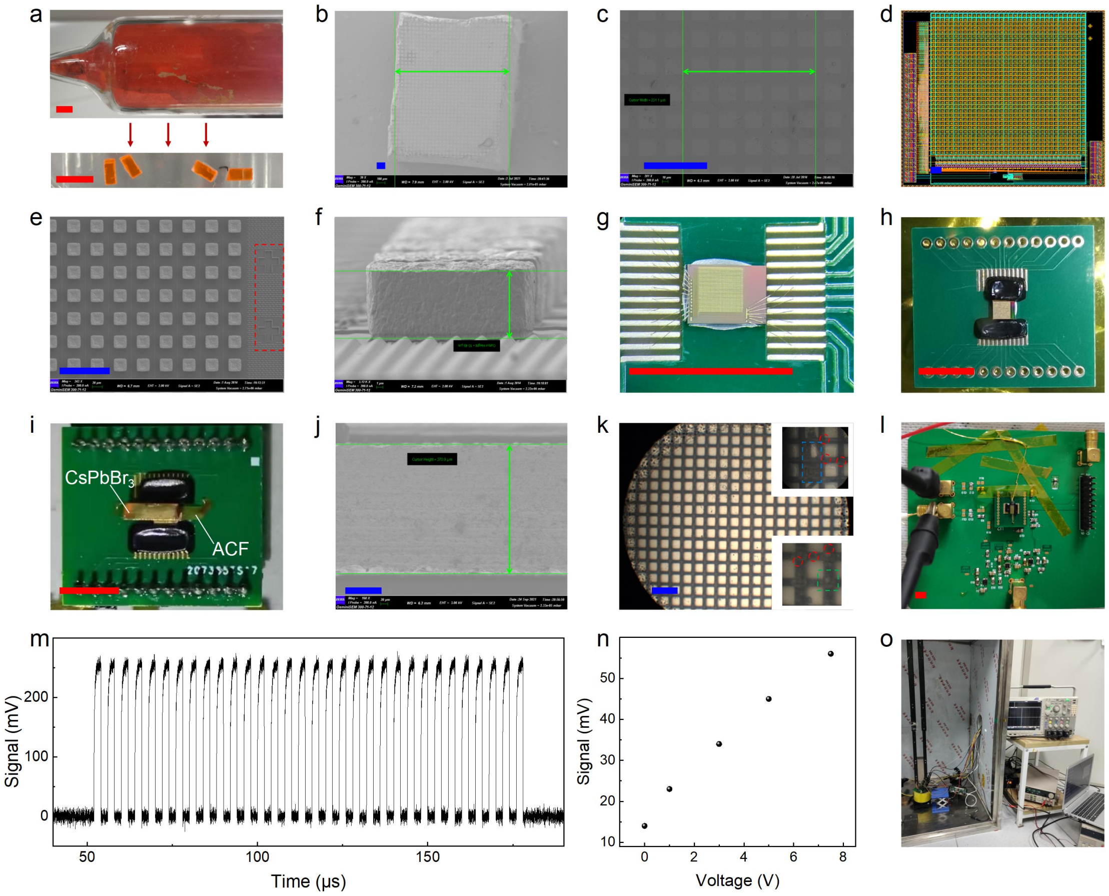

Supplementary Figure 9. Pictures of the heterogeneous integration process and result. (a) Optical photos of the Bridgman's grown ingot and the perovskite single crystal (PSC) samples after cutting and polishing. (b-c) PSC with pixel electrodes on the surface. The size of a single pixel is 46×46 μm^2, and its electrode size is 30×30 μm^2, which are all complete, uniform in size, and have little evaporation shadows. (d) The complementary metal-oxide-semiconductor (CMOS) layout, corresponds to the optical photo in Figure 3f. (e-f) CMOS die with under bump metal (UBM). The UBM is made of gold and has a height of 10 μm, which is enough to squeeze the conductive particles in the anisotropic conductive tape (ACF). Alignment male keys are reserved, as shown in the red box. (g-h) The pins of the CMOS die are connected to the lead-out electrodes on the sub-PCB. Since the gold wire is very fragile, it needs to be protected by resin encapsulation. (i-k) Connecting the 370 μm thick PSC and CMOS using ACF flip-chip bonding. We can observe whether ACF is connected well in the optical photograph, by looking from above on the PSC without electrodes. The illustration shows dead pixels, the Au/Ni particles, and the aligned UBM underneath.

The optical photo without dead pixels is shown in Figure k. (l) Enlarge image of Figure 3f. (m) The electrical signals scanned and outputted in a specific row. (n) The signals under different bias voltages. (o) Photo of the testing system. During the measurement, the CMOS circuit is adjustably biased by a direct-current power with an oscilloscope observing the signal waveforms at various contact points, and a computer controls the operation of the X-ray tube. The scale bars in red color are 5 mm, and in blue color are 100 μm. The green scale in Figure b is 1573 μm, in Figure c is 231.1 μm, in Figure f is 10.49 μm, and in Figure j is 370.9 μm.

Source data are provided as a Source Data file.

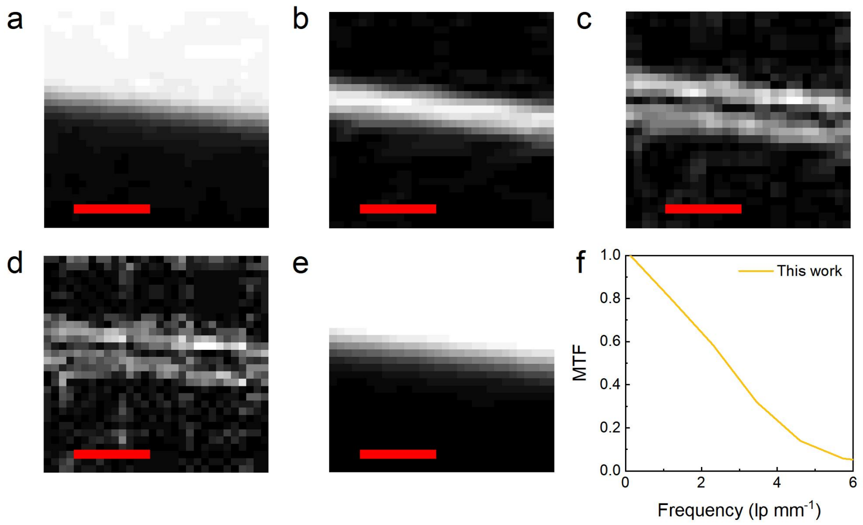

Supplementary Figure 10. The result of imaging in reality and the simulation results. (a) X-ray imaging of the blade edge with the 32×32 CMOS-based detector. (b-e) Simulation results via the image stylization, respectively represent Sobel, Laplacian of the Gaussian, Laplacian, and mean filtering convolution kernels, according to Figure 4 in the manuscript. All scale bars in red color are 500 μm. (f) The modulation transfer function (MTF) of Figure a, compared with the work[8]. It shows our CMOS integrated detector having an MTF of 4.2 lp mm^{-1} at 20%, better than the perovskite-film-TFT-integrated detector. TFT represents thin-film transistor, CMOS represents complementary metal-oxide-semiconductor.

We have demonstrated convolution kernel-based intelligent imaging through PCB-level circuits, and demonstrated CMOS-based detector imaging through

integration. The simulation here envisions the effects of full-on-chip integrated in-X-ray-detetor computing. PCB represents printed circuit board.

Source data are provided as a Source Data file.

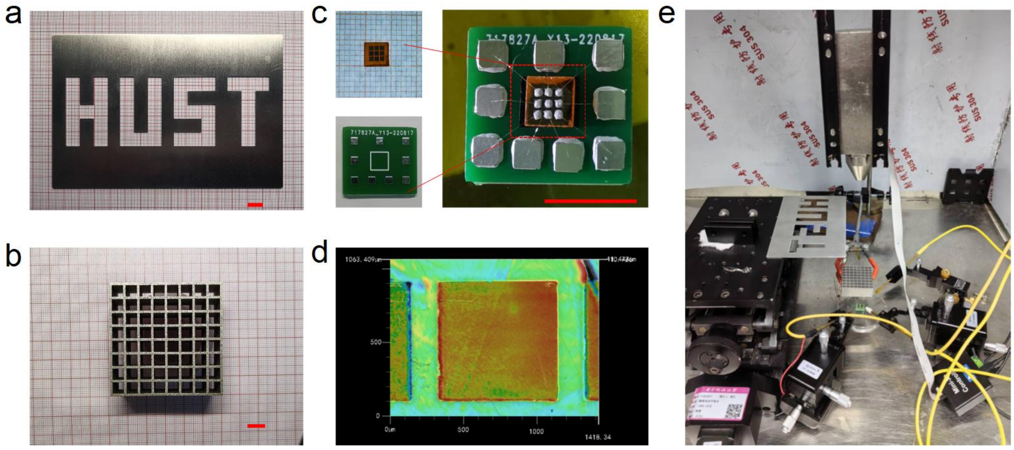

Supplementary Figure 11. The imaging setup for edge extraction. (a) Optical photo of the object. (b) Photo of the collimator. (c) Photo of the detector. There are 3×3 sub-pixel electrodes on the $CsPbBr_3$ perovskite single crystal (PSC), with an electrode size of 0.8 mm and a space of 0.2 mm. The top sub-pixel electrodes are connected to the printed circuit board (PCB) and are applied with different bias voltages through the electrometer. The bottom common electrode collects the summed signal for the macro pixel. To achieve the Laplacian kernel effect, we connect the 8 surrounding sub-pixels on the back of the PCB by silver paste and apply the same reverse bias voltage. In contrast, the central sub-pixel was applied a forward voltage. This design facilitates the implementation of edge extraction imaging or other effects in board-level circuits. (d) Confocal laser scanning microscope (CLSM) photo of the sub-pixels. (e) Photos of the test system. A displacement stage moves the object for imaging. All scale bars in red color are 1 cm.

Furthermore, Kirchhoff's current law states that at any node in a circuit, the algebraic sum of the currents is zero. Here, the electrometer's collection probe collects the signal current of the carbon electrode. Mathematically, its value is the sum of the currents of each sub-pixel, with the opposite vector of the sum.

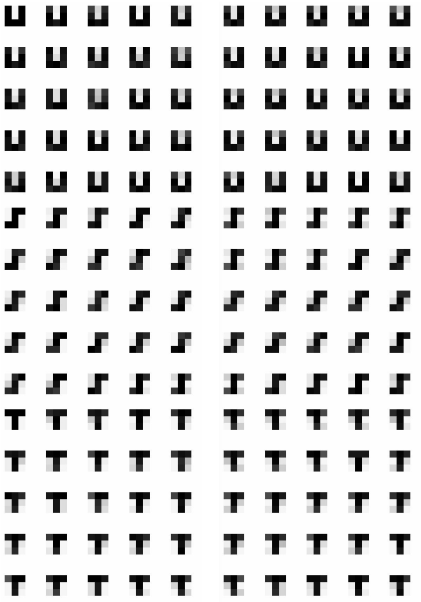

Supplementary Figure 12. Sampling data for other iron sheets. These iron sheets, in the shape of U, S, and T letters, were also placed according to the H-type letter iron sheet in Figure 5b, with varying horizontal displacements and rotation angles from the original positions. The above data together constituted the dataset.

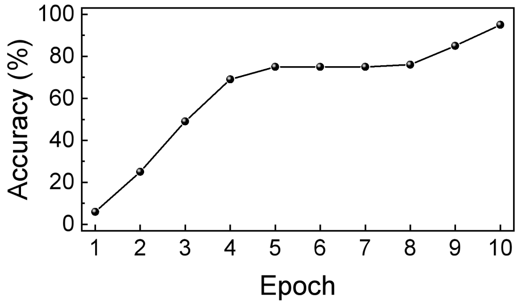

Supplementary Figure 13. The accuracy for another calculation process with other different initial values. It was also close to 100% after several epochs. The output values for this process can be seen in Supplementary Figure 14.

Source data are provided as a Source Data file.

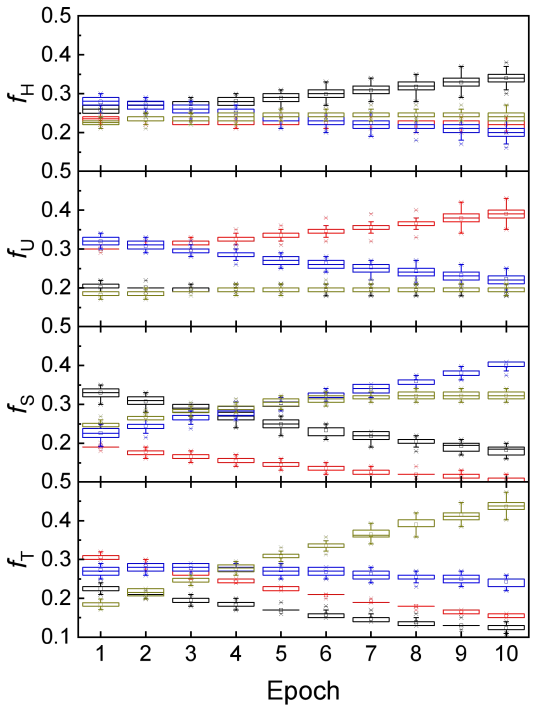

Supplementary Figure 14. The detailed evolution process for another calculation process with other different initial values. The four boxplots from top to bottom represent the processing results of the four feature convolution kernels. The grid lines of the box are divided according to 25%, 50% and 75% of the data. Percentiles are represented by horizontal lines for minimum and maximum values, crosses for 1% and 99%, and boxes for mean values. The colors black, red, blue, and yellow represent the letters H, U, S, and T, respectively.

Source data are provided as a Source Data file.

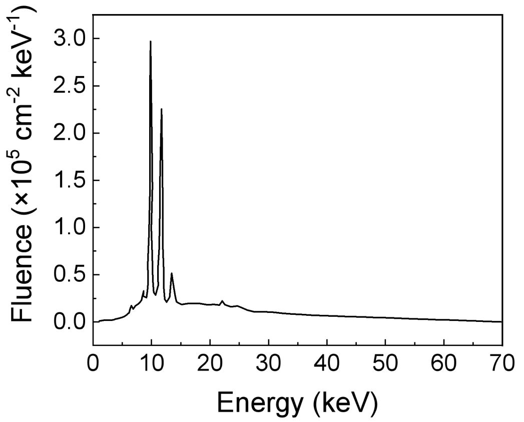

Supplementary Figure 15. The incident X-ray fluence with the gold-target tube in the article. Source data are provided as a Source Data file.

Table S1. The parameters of Computed Tomography products of various companies. The following rankings are in no particular order. The month of some information is unknown, only the year is given. CT represent Computed Tomography.

Company	CT product	Related Information	
		The maximum amount of data rate generated (Mcps)	Release time
United Imaging	uCT 960+	35,800	2020
	uCT 780	7,250	2018
	uCT 550	2,000	2017
	uCT 510	301	2013
GE	Revolution CT	40,000	2014
	Discovery HD 750	13,800	2010
	Optima 660	2,787	2011
	BrightSpeed 16	318	2009
	LightSpeed 16	53	2002
Siemens	SOMATOM Force	21,480	2013
	SOMATOM Definition	3,000	2005
	Sensation 64	515	2003
	Spirit	84	2005
Toshiba	Aquilion One 320	12,700	2011
	Aquilion 16	416	2012
Philips	IQon	25,860	2017
	Brilliance iCT	8,600	2009
	Ingenuity core	4,320	2014
Neusoft	NeuViz Epoch	27,540	2020
	NeuViz Prime	5,160	2017
	NeuViz 128	3,590	2015
	NeuViz 64	1,790	2012

Table S2. The parameters of Computed Tomography slip-ring products. The month of some information is unknown, only the year is given in the table below. CT represent Computed Tomography.

Company	Related Information of the CT slip-ring product	
	The maximum transmission data rate (Mbps)	Release time
**Schleifring (Germany)**	10,000	2018
	50~10,000	2012
	600~3,700 (typical value 2,500)	2003
**Moog (U.S.)**	10,000	2022
	1,000~10,000 (typical value 5,000)	2018
	3,000~7,000 (typical value 1,250)	2005

Table S3. Glow discharge mass spectrometry data about the purity of the $CsPbBr_3$ PSC. By gradually shortening the melting zone and reducing the speed of the zone-melting process, we obtained high-purity raw materials and applied them to single-crystal growth. The results tabulated with a “<” sign are detection limits. The following table records the concentration information of 72 elements in the sample. For most of the elements, their concentration lies below the detection limits. The total impurity level determined, excluding the elements outside the detection limit, is about 1 ppm wt. (μg g^{-1}). That is, the purity is approximately 99.9999%.

Elements	Concentration (ppm) wt.	Elements	Concentration (ppm) wt.	Elements	Concentration (ppm) wt.
Li	<0.050	Ge	<0.50	Sm	<0.050
Be	<0.050	As	<0.050	Eu	<0.050
B	<0.050	Se	<0.50	Gd	<0.050
F	<0.050	Rb	0.72	Tb	<0.050
Na	0.14	Sr	<0.050	Dy	<0.050
Mg	<0.050	Y	<0.050	Ho	<0.050
Al	0.12	Zr	<0.050	Er	<0.050
Si	0.22	Nb	<0.050	Tm	<0.050
P	<0.050	Mo	<0.050	Yb	<0.050
S	0.08	Ru	<0.050	Lu	<0.050
Cl	<1.0	Rh	<0.050	Hf	<0.050
K	<0.10	Pd	<0.050	Ta	<0.050
Ca	0.18	Ag	<0.50	W	<0.050
Sc	<0.050	Cd	<0.50	Re	<0.050
Ti	<0.050	Sn	<0.50	Os	<0.050
V	<0.050	Sb	<0.10	Ir	<0.050
Cr	<0.050	Te	<0.10	Pt	<0.050
Mn	<0.050	I	0.26	Au	<0.050
Fe	<0.050	Cs	Matrix	Hg	<0.050
Co	<0.050	Ba	<0.050	Tl	<0.050
Ni	<0.050	La	<0.050	Pb	Matrix
Cu	<0.050	Ce	<0.050	Bi	<0.050
Zn	<0.050	Pr	<0.050	Th	<0.050
Ga	0.14	Nd	<0.050	U	<0.050

Supplementary Table 4. Response time comparison of perovskite single crystal X-ray detectors.

Materials	Thickness and bias voltage	kVp for X-ray	Response / decay time	Reference
$Cs_2AgBiCl_6$	2 V	30	< 3 ms	9
$Cs_2AgBiBr_6$	3 V mm^{-1}	50	0.77 ms	10
$(MA)_3Bi_2I_9$	60 V mm^{-1}	40	23.3 / 31.4 ms	11
$(MA)_3Bi_2I_9$	286 V mm^{-1}	40	0.266 / 0.417 ms	12
$(NH_4)_3Bi_2I_9$	5 V mm^{-1}	8	5 ms	13
$(BDA)PbI_4$	310 V mm^{-1}	40	7.3 / 22.5 ms	14
$(BA)_2PbBr_4$	0.2 V	50	307 / 98 ms	15
$(BA)_2PbI_4$	10 V mm^{-1}	30	4.5 / 4.3 ms	16
$(FA)_{0.55}(MA)_{0.45}PbI_3$<br>$MAPbI_3$	0 V	50	0.086 / 0.5 ms<br>0.98 / 1.02 ms	17
$MAPbBr_3$	0.5 V mm^{-1}	8	0.255 ms	18
$MAPbBr_3$	0.05 V mm^{-1}	22	0.73 ms	19
CsFAGA:Sr $PbBr_{0.3}I_{2.7}$<br>CsFAGA $PbBr_{0.3}I_{2.7}$<br>CsFA $PbBr_{0.3}I_{2.7}$	0.1 V mm^{-1}	60	880 / 750 ms<br>940 / 870 ms<br>1120 / 1070 ms	20
Ours, $CsPbBr_3$	0.4 V mm^{-1}	50	2.89 / 2.50 ms	This work

Supplementary Table 5. Response time comparison of perovskite single crystal visible photo-detectors.

Materials	Thickness and bias voltage	Light source	Response / decay time	Reference
$FA_{0.55}MA_{0.45}PbI_3$	1 V	870 nm	34 / 164 μs	21
$(BA)_n(MA)_{n-1}Pb_nI_{3n+1}$	1 V	Xenon lamp	1.7 / 3.9 μs	22
			23 / 19 μs	
			0.25 / 1.75 ms	
			383 / 177 ms	
			773 / 385 ms	
$CsPbBr_3$	0 V	550 nm	230 / 60 ms	23
$MAPbI_3$	60 V mm^{-1}	532 nm	470 / 530 μs	24
	100 V mm^{-1}		420 / 470 μs	
	140 V mm^{-1}		420 / 460 μs	
$MAPbBr_3$	450 μm at 0 V	355 nm	8 / 613 μs	25
$MAPbCl_3$	450 μm at 1 V		25 / 940 μs	
$MAPbBr_3$	450 μm at 1 V		19 / 860 μs	
$(BA)_2(MA)_2Pb_3I_{10}$		365 nm	0.5 / 20-60 μs	26
$(PEA)_2PbBr_4$			410 / 310 μs	
	72 V mm^{-1}	380 nm	0.147 / 0.768 μs	
LDSC-$MAPbBr_3$	2.02 mm at 0 V	White light	93 μs	27
	2.02 mm at 4 V		62 μs	
HT-$MAPbBr_3$	1.42 mm at 0 V		206 μs	
	1.42 mm at 4 V		205 μs	
$MAPbBr_3$	1 mm at 0.1 V	470 nm	216 μs	19
	2.6 mm at 0.1 V		1300 μs	
Ours, $CsPbBr_3$	0.4 V mm^{-1}	365	40 / 710 μs	This work

Supplementary Table 6. Comparison chart of perovskite X-ray detector.

Active layer	Intelligent imaging	Chip-integrated	LDR[a] (dB)	Sensitivity (μC Gy_{air}^{-1} cm^{-2})	Size	Response time	Stability	Reference
$(NH_4)_3Bi_2I_9$	×	×	20	Parallel: 8.4×10^4<br>Perpendicular: 803	SC[b]: 1~2 cm	Parallel: 13 ms<br>Perpendicular: 5 ms	NA[c]	13
$Cs_3Bi_2I_9$	×	×	20	1652	Image: 7.5 cm<br>SC: 0.5-1 mm^2	NA	Keep stable under bias and radiation	28
FPEA SC	×	×	26	3402	SC: 1×1 cm^2	0.8 μs, fitting the transient photocurrent	Keep stable under bias and radiation	29
BAMA film	×	×	84	0.276×10^6	NA	0.5 / 20-60 μs	Keep stable under bias and radiation	30
2D/3D film	×	×	71	1.95×10^4	Image: 5.1×5.1 cm^2	X-ray: 216 / 174 ms<br>233 / 182 ms	Keep stable under bias and radiation	31
$MAPbBr_3$ SC	×	×	36	8.4×10^4	SC: 1×1 cm^2	390 nm: 23 μs	Keep stable	32
$MAPbBr_3$ SC	×	×	77	2.1×10^6	Image: 1.5 cm<br>SC: 5.8×5.8 mm^2	255 μs	NA	18
$CsPbI_2Br$ film	×	×	24	1.2×10^6	12×12 cm^2	X-ray: 769 ns (1 μm)	Keep stable under radiation	33
$CsPbBr_3$ SC	×	×	90	15 to 5111 (depends)	Ingot: 1 inch<br>SC: 5×5 mm^2	γ: 2.6 μs at 300 V	Keep stable under bias and radiation	34
$CsPbBr_3$ SC	√	√	106	396	Ingot: 1 inch<br>SC: 5×5 mm^2	X-ray: 2.89 / 2.50 ms<br>365 nm: 30 / 710 μs<br>γ: 4 μs at 800 V	Keep stable under bias and radiation	This work

(a) LDR means linear dynamic range.

(a) SC means single crystal.

(b) NA means not applicable.

Supplementary Note 1. The amount of data generated by X-ray detectors.

For Computed Tomography (CT), the maximum amount of data rate detected is determined using the following formula (1),

$$\text{Rate}_{\text{max}} = \frac{P \cdot n \cdot m \cdot v \cdot f}{a \cdot b} \quad (1)$$

where P is the number of slices (the number of images acquired simultaneously by the CT data acquisition system), n is the number of single-slice CT detectors, m is the number of data bits, v is the rotation speed, f is the sampling frequency, and $a \times b$ represents the reconstruction image resolution. General Electric, Philips, and Siemens (known as G. P. S.) companies offer various types of CT products, and they have introduced low-configuration products to compete in the low-end market. Here, two other companies, United-imaging and Neusoft, with less representative products are also introduced as references, which can better illustrate the overall development speed and parameter evolution in the field. The detailed parameters of their CT products can be found in Supplementary Table 1 and the data rates are marked in red and listed in Figure 1b. Products from the same company use symbols with the same fill status. The red straight line qualitatively represents the development trend of CT data generated by X-ray detectors, which shows exponential growth.

Besides, the main two suppliers (Schleifring from Germany and Moog from the U.S.) of CT slip-ring manufacturers, which have over 95% market share, are presented in Figure 1b. The CT slip ring powers the X-ray tube and detector and outputs data, and its transmission rate is listed by blue patterns in Supplementary Figure 1b, represented by one type slip ring, the coupled rotary joints. The blue straight line qualitatively represents the development trend. So far, they have increased the data transmission capacity of capacitively coupled slip rings to more than 10,000 Megabits per second (Mbps). However, its growth rate, limited by electron mobility, is much slower than that of detectors. Efforts have been made to enhance the transmission rate of the slip ring using methods such as photoelectric coupling, geometric optical elements design, optical folding in off-axis space, and multiplexing technologies. The detailed parameters of their slip-ring products can be

found in Supplementary Table 2. All the above-mentioned information can be found or calculated from the products' datasheet or the companies' public reports.

Similarly, dynamic digital radiography (DR), as a very important representative application, also generates a significant amount of data. The Mercu 1717V3 product from Shanghai Iray Company, for example, has a transmission rate of approximately 1,000 Mbps. It can operate normally at low frame rates, such as 5 frames per second (fps), but it needs to combine adjacent pixels (2×2 or 3×3) to output one value together at high frame rates like 20 or 30 fps. This indicates that the data transmission rate limits the dynamic digital radiography detectors, which cannot be compatible with both high spatial resolution and high dynamic frame rate. Furthermore, this hinders its development towards larger imaging areas, smaller pixels, and real-time imaging.

Finally, we used the orange straight line in Supplementary Figure 1b to represent the detected data rate with edge extraction, which is estimated by using the compression rate of about 50% in the manuscript.

Supplementary Note 2. Thermally Stimulated Current and the Simultaneous Multiple Peak Analysis.

The Thermally Stimulated Current (TSC) technology is a straightforward and effective method for analyzing the energy levels and densities of the defects in semiconductors with high resistivity[35,36]. To ensure that the defect levels could capture enough free photogenerated carriers, we first cooled the devices in the Cold Trap using liquid nitrogen and then irradiated the device for 5 min using the X-ray source, as shown in Supplementary Figure 15. Next, we applied a bias voltage of -10 V and waited for the electrometer's current reading to stabilize before starting the temperature rise. With the temperature increased in the dark from 88 to 290 K at a constant heating rate (0.10 K s^{-1}), the trapped electrons and holes were released by thermal emission, and the TSC spectrum was obtained by the Keithley 6517B Source Meter.

The Simultaneous Multiple Peak Analysis (SIMPA) method is an analytical procedure for the simultaneous and complete characterization of the traps found in the examined semiconductor material[37,38]. It has been used to characterize traps in perovskites[36,39]. SIMPA is based on the assumption that the TSC spectrum is a sum of TSC peaks belonging to the specific traps and the dark current $I_{\mathrm{dark}}(T)$=C exp($-E_{\mathrm{trap}}/kT$), where C is a constant and E_{trap} is the trap level analogous to the mid-gap level in the semiconductor. The trap level plays an important role in electrical compensation and the strength of the dark current. Since the resistivity of our PSC device was on the order of 10^9 Ω cm, one can infer that electrical compensation was achieved. The temperature-dependent fitting function $I_{\mathrm{SIMPA}}(T)$, including the sum of all features of the TSC spectrum is given by formula (2),[37]

$$I_{\mathrm{SIMPA}}(T) = \sum_{i=1}^{m} I_{\mathrm{TSC}}^{i}(T) + I_{\mathrm{dark}}(T) \tag{2}$$

where $I_{\mathrm{TSC}}^{i}(T)$ represents the ith individual TSC peak and m is the total number of different traps calculated. We regard $I_{\mathrm{SIMPA}}(T)$ as the result of the superposition of multiple I_{TSC} using the theoretical TSC function, where each I_{TSC} represents a certain defect and its parameters. In detail, the ith individual TSC peak can be described as[37],

$$I_{\mathrm{TSC}}(T) = K_{\mathrm{G}}\mu\, N\tau\, D_{\mathrm{t}}T^2 \exp[-\frac{E_{\mathrm{a}}}{kT} - \frac{kD_{\mathrm{t}}}{\beta E_{\mathrm{a}}}T^4 \mathrm{e}^{-E_{\mathrm{a}}/kT} \times (1 - 4\frac{kT}{E_{\mathrm{a}}} + 20\frac{k^2T^2}{E_{\mathrm{a}}^2})] \quad (3)$$

where K_G denotes a geometrical factor and N is the density of the filled certain trap at the beginning of the temperature ramp, variable E_a is the activation energy of the certain trap, β is the heating rate, and D_t is the temperature independent and trap dependent coefficient that includes the electrons or holes capture cross section. The formula above is used as the fitting function, with E_a, D_t and N as unknowns. Here, we only focus on N, which is associated with the height, position, and width of the individual TSC peak, respectively.

First, we can calculate the effective collected charge Q released from this defect level[39], which is the integral of I_{TSC} and can be expressed as formula (4),

$$Q = \int I_{\mathrm{TSC}}(t)\, \mathrm{d}t = \frac{1}{\beta}\int_{T_0}^{T} I_{\mathrm{TSC}}(T)\, \mathrm{d}T \quad (4)$$

In the formula, $\mathrm{d}T=\beta \mathrm{d}t$. We then calculate the concentration of this defect level using the following formula (5),

$$N = \frac{Q}{V_{\mathrm{eff}} \cdot \mathrm{e} \cdot G} \quad (5)$$

where V_{eff} is the effective irradiated volume of the sample, G is the collection efficiency of carriers, also given by the Hecht formula, $G=\mu\tau V/L^2$. In the SIMPA operations in this article, we calculated the defect concentrations of adjacent individual TSC peaks together to reduce the influence of various unideal factors. Although this would make the concentration of defects appear higher. For more detailed SIMPA results of the PSC device, readers can refer to Supplementary Figure 4.

Supplementary Note 3. Simulation of X-ray detection sensitivity.

Sensitivity, S, is an important parameter for direct X-ray detectors, as it reflects the charge generated by the semiconductor per unit area (cm^2) per unit dose (Gy or R). The sensitivity is related to the carrier formation energy (or pair creation energy), $W_{\pm}$, and charge collection efficiency (CCE, η_{cc}) of the semiconductor. When an X-ray photon with energy, E_{en}, is totally absorbed, the semiconductor will create $E_{en}/W_{\pm}$ electron hole (e-h) pairs. If the CCE is 100%, which means that all generated charges are collected by electrodes, the maximum sensitivity of the detector is given by[40,41],

$$S_0(E_{en}) = \left[\frac{5.45 \times 10^{13}\ \mathrm{e}}{(\alpha_{air}/\rho_{air})W_{\pm}}\right](\frac{\alpha_{en}}{\alpha}) \tag{6}$$

where α_{air} is the energy absorption coefficient of air, ρ_{air} is the density of air, α_{en} is the energy absorption coefficient of the semiconductor.

Assuming that the top electrode is negatively biased, the CCE of the detector can be derived from the Hecht equation and is given by formula (7),

$$\eta_{cc} = l_e\left[1 + \frac{1}{\eta(\Delta/l_e - 1)}(\mathrm{e}^{-\frac{1}{l_e}} - \mathrm{e}^{-\frac{1}{\Delta}})\right] + l_h\left[1 - \frac{1}{\eta(\Delta/l_h + 1)}(1 - \mathrm{e}^{-\frac{1}{\Delta}-\frac{1}{l_h}})\right] \tag{7}$$

where $l_{e,h} = \mu_{e,h}\tau_{e,h}E/L$ ($\mu_{e,h}$ is the carrier mobility, $\tau_{e,h}$ is the carrier lifetime and E is the electron field) is the normalized electron and hole schubweg, and $\Delta = 1/\alpha L$ is the normalized attenuation depth. Hence, the CCE and absorption-limited X-ray sensitivity of the detector can be given by formula (8),

$$S(E_{en}) = \eta(E_{en})\eta_{cc}(E_{en})S_0(E_{en}) \tag{8}$$

For a poly-energetic X-ray beam, the sensitivity of the detector is,

$$S = \int_{E_{en_{min}}}^{E_{en_{max}}} \eta(E_{en})\eta_{cc}(E_{en})S_0(E_{en})\frac{\Phi(E_{en})}{\Phi_0}\mathrm{d}E_{en} \tag{9}$$

Where Φ is the incident X-ray fluence with a unit of photons per unit area per unit energy. For the numerical simulations in the article, we adopted the parameters for the $CsPbBr_3$ single crystal, a $\mu\tau$ value of 2.05×10^{-3} cm^2 V^{-1}, a bias voltage of 100 V, a thickness of 2 mm, a $W_{\pm}$ value of 5.4 eV, a density of 4.85 g cm^{-3}, and an energy spectrum of the simulated gold target X-ray tube under a driving voltage of 70 kVp and a working current of 140 μA, as shown in Supplementary Figure 15.

Supplementary Note 4. Design methodology of the bias voltages.

According to the mathematical definition of the Laplacian kernel, the photo-current should be -1:8 between the surrounding sub-pixel and the central sub-pixels, and the sum of the photo-current for the macro-pixel should be zero. We made slight adjustments based on the *I-V* and *I-t* data as references. Finally, in Supplementary Figure 11, we applied -73 V and 95 V biases, which resulted in a photo-response current ratio of -1:8. At this point, the bias voltage is not -1:8 due to the rectifying effect of the N-I-P detector.

In reality, the two carrier collection capabilities of the detector are not completely identical. Under the same X-ray intensity, it would have different on-off ratios, as shown in Supplementary Figure 5c. The linear dynamic range (LDR) will be reduced accordingly. In fact, the larger the difference in carrier collection capabilities, the higher the dark current of the macro-pixel detector. To achieve a high LDR and good in-X-ray-detector computing performance, the material should have more balanced charge collection capabilities.

Supplementary Note 5. Additional notes on the linear dynamic range.

The detector in Figure 3 features a stable trap-free device structure and achieves a high linear dynamic range of 106 dB, exceeding state-of-the-art X-ray detectors. Here, we only make comparisons with X-ray detection rather than visible light detectors due to the different carrier relaxation processes.

In addition, we must also explain the importance of ultra-high linear dynamic range for in-X-ray-detector computing devices. Detectors with low linear dynamic range (LDR) have different sensitivities when overexposed or underexposed. The photo-current of sub-pixels should have been at the designed ratio, but this ratio changed at this time. The nonlinear response cannot achieve a zero response signal when the sub-pixel signals are summed. Therefore, the effect of the convolution kernel cannot be achieved.

Supplementary References.

1. Toufanian, R., Swain, S., Becla, P., Motakef, S., & Datta, A. Cesium lead bromide semiconductor radiation detectors: crystal growth, detector performance and polarization. *J. Mater. Chem. C* **10**, 12708-12714 (2022)

2. He, Y. et al. Demonstration of energy-resolved γ-ray detection at room temperature by the $CsPbCl_3$ perovskite semiconductor. *J. Am. Chem. Soc.* **143**, 2068-2077 (2021)

3. Chen, S. et al. Identifying the soft nature of defective perovskite surface layer and its removal using a facile mechanical approach. *Joule* **4**, 2661-2674 (2020)

4. Owens, A. Semiconductor materials and radiation detection. *J. Synchrotron Radiat.* **13**, 143-150 (2006)

5. Greiffenberg, D., Fauler, A., Zwerger, A., & Fiederle, M. Energy resolution and transport properties of CdTe-Timepix-Assemblies. *J. Instrum.* **6**, C01058 (2011)

6. Kasap, S. O., Koughia, K. V., Fogal, B., Belev, G., & Johanson, R. E. The influence of deposition conditions and alloying on the electronic properties of amorphous selenium. *Semiconductors* **37**, 789-794 (2003)

7. Li, H., Niu, G., Nie, Z., Tang, J., & Liu, D. A CMOS Readout Circuit with Low Detection Limit and High Linearity for Perovskite-based Direct X-ray Detector. In 15th *Int. Conf. Solid-State Integr. Circuit Technol.* (ICSICT) 1-3 (IEEE 2020)

8. Deumel, S. et al. High-sensitivity high-resolution X-ray imaging with soft-sintered metal halide perovskites. *Nat. Electron.* **4**, 681-688 (2021)

9. Naveen, K. T. et al. Self-Powered X-ray Detection and Imaging using $Cs_2AgBiCl_6$ Lead-Free Double Perovskite Single Crystal. *ACS Appl. Electron. Mater.* **4**, 4530–4539 (2022)

10. Pan, W. et al. $Cs_2AgBiBr_6$ single-crystal X-ray detectors with a low detection limit. *Nat. Photon.* **11**, 726–732 (2017)

11. Liu, Y. et al. Inch-Size 0D-Structured Lead-Free Perovskite Single Crystals for Highly Sensitive Stable X-Ray Imaging. *Matter* **3**, 180–196 (2020)

12. Liu, Y. et al. Large Lead-Free Perovskite Single Crystal for High-Performance Coplanar X-Ray Imaging Applications. *Adv. Opt. Mater.* **8**, 2000814 (2020)

13. Zhuang, R. et al. Highly sensitive X-ray detector made of layered perovskite-like $(NH_4)_3Bi_2I_9$ single crystal with anisotropic response. *Nat. Photon.* **13**, 602–608 (2019)

14. Shen, Y. et al. Centimeter-Sized Single Crystal of Two-Dimensional Halide Perovskites Incorporating Straight-Chain Symmetric Diammonium Ion for X-Ray Detection. *Angew. Chem. Int. Ed.* **59**, 14896. (2020)

15. Xu, X. et al. Two-Dimensional Perovskite Single Crystals for High-Performance X-ray Imaging and Exploring MeV X-ray Detection. *Energy Environ. Mater.* e12487 (2023)

16. Joydip, G. et al. Efficient and Highly Stable X-ray Detection and Imaging using 2D $(BA)_2PbI_4$ Perovskite Single Crystals. *ACS Photonics* **9**, 3529-3539 (2022)

17. Wu,J. Self-Powered $FA_{0.55}MA_{0.45}PbI_3$ Single-Crystal Perovskite X-Ray Detectors with High Sensitivity. *Adv. Funct. Mater.* **32**, 2109149 (2022)

18. Wei, W. et al. Monolithic integration of hybrid perovskite single crystals with heterogenous substrate for highly sensitive X-ray imaging. *Nat Photon.* **11**, 315–321 (2017)

19. Wei, H. et al. Sensitive X-ray detectors made of methylammonium lead tribromide perovskite single crystals. *Nat Photon.* **10**, 333–339 (2016)

20. Jiang, J. et al. Synergistic strain engineering of perovskite single crystals for highly stable and

sensitive X-ray detectors with low-bias imaging and monitoring. *Nat. Photon.* **16**, 575–581 (2022)
21. Li, W., Rao, H., Chen, B., Wang, X., & Kuang, D. B. et al. A formamidinium–methylammonium lead iodide perovskite single crystal exhibiting exceptional optoelectronic properties and long-term stability. *J. Mater. Chem. A* **5**, 19431-19438 (2017)
22. Wang, K., Wu, C., Yang, D., Jiang, Y., & Priya, S. et al. Quasi-two-dimensional halide perovskite single crystal photodetector. *ACS nano* **12**, 4919-4929 (2018)
23. Saidaminov, M. et al. Inorganic lead halide perovskite single crystals: phase-selective low-temperature growth, carrier transport properties, and self-powered photodetection. *Adv. Opt. Mater.* **5**, 1600704 (2017)
24. Song, Y. et al. Elimination of interfacial-electrochemical-reaction-induced polarization in perovskite single crystals for ultrasensitive and stable X-ray detector arrays. *Adv. Mater.* **33**, 2103078 (2021)
25. Yan, J. et al. Controllable Perovskite Single Crystal Heterojunction for Stable Self-Powered Photo-Imaging and X-Ray Detection. *Adv. Opt.l Mater.* **10**, 2200449 (2022)
26. Lédée, F. et al. Ultra-Stable and Robust Response to X-Rays in 2D Layered Perovskite Micro-Crystalline Films Directly Deposited on Flexible Substrate. *Adv. Opt. Mater.* **10**, 2101145 (2022)
27. Yao, F. et al. Room-temperature liquid diffused separation induced crystallization for high-quality perovskite single crystals. *Nat. Commun.* **11**, 1194 (2020)
28. Zhang, Y. et al. Nucleation-controlled growth of superior lead-free perovskite $Cs_3Bi_2I_9$ single-crystals for high-performance X-ray detection. *Nat. Commun.* **11**, 2304 (2020)
29. Li, H. et al. Sensitive and stable 2D perovskite single-crystal X-ray detectors enabled by a supramolecular anchor. *Adv. Mater.* **32**, 2003790 (2020)
30. Tsai, H. et al. A sensitive and robust thin-film x-ray detector using 2D layered perovskite diodes. *Sci. Adv.* **6**, eaay0815 (2020)
31. Xu, X. et al. Sequential Growth of 2D/3D Double-Layer Perovskite Films with Superior X-Ray Detection Performance. *Adv. Sci.* **8**, 2102730 (2021)
32. Wei, H. et al. Dopant compensation in alloyed $CH_3NH_3PbBr_{3-x}Cl_x$ perovskite single crystals for gamma-ray spectroscopy. *Nat. Mater.* **16**, 826-833 (2017)
33. Lai, P. T. et al. All-Vacuum-Deposited Perovskite X-ray Detector with a Record-High Self-Powered Sensitivity of 1.2 C Gy^{-1} cm^{-3}. *ACS Appl. Mater. Interfaces* **14**, 19795-19805 (2022)
34. He, Y. et al. Sensitivity and Detection Limit of Spectroscopic-Grade Perovskite $CsPbBr_3$ Crystal for Hard X-Ray Detection. *Adv. Funct. Mater.* **32**, 2112925 (2022)
35. Guo, R. et al. Influence of deep level defects on carrier lifetime in CdZnTe. *J. Appl. Phys.* **117**, 094502 (2015)
36. He, Y. et al. High spectral resolution of gamma-rays at room temperature by perovskite $CsPbBr_3$ single crystals. *Nat. Commun.* **9**, 1609 (2018)
37. Pavlović, M., & Desnica, U. V. Precise determination of deep trap signatures and their relative and absolute concentrations in semi-insulating GaAs. *J. Appl. Phys.* **84**, 2018-2024 (1998)
38. Desnica, U. V., Pavlović, M., Fang, Z. Q., & Look, D. C. Thermoelectric effect spectroscopy of deep levels in semi-insulating GaN. *J. Appl. Phys.* **92**, 4126-4128 (2002)
39. Zhang, M. et al. Determination of defect levels in melt-grown all-inorganic perovskite $CsPbBr_3$ crystals by thermally stimulated current spectra. *J. Phy. Chem. C* **122**, 10309-10315 (2018)

40. Panneerselvam, D. M., & Kabir, M. Z. Evaluation of organic perovskite photoconductors for direct conversion X-ray imaging detectors. J. Mater. *Sci-Mater. El.* **28**, 7083-7090 (2017)
41. Kasap, S. O. X-ray sensitivity of photoconductors: application to stabilized a-Se. *J. Phys. D Appl. Phys.* **33**, 2853 (2000)
