# Supplementary material for: Reconfigurable perovskite X-ray detector for intelligent imaging
Source: Nat Commun. 2024 Feb 27;15:1769. doi: 10.1038/s41467-024-46184-0 (PMC10899650; doi:10.1038/s41467-024-46184-0)
Supplement: Supplementary file 3 — Description of Additional Supplementary Files [file 41467_2024_46184_MOESM3_ESM.pdf]

Description of Additional Supplementary Files

Supplementary Software 1

Python 3.6 or higher and TensorFlow 2.0 or higher are required.
The image_3D.mat files has shape of (width, height,#images) , image_address.mat files has shape of (#images)

Supplementary Data 1

The X-ray images including Baggages-0002-0001 in this study are provided in the public GDXray+ database, under accession website. On the site, all X-ray images are available at the Download tab.
The self-made database for convolutional neural networks, including all letter images used in Figure 5, are provided in the attachment Supplementary Data 1. The sub-file names, h, u, s and t, respectively, represent the corresponding imaging objects.
