# Supplementary figures and images for: Reconfigurable perovskite X-ray detector for intelligent imaging (part 2 of 2)
Source: Nat Commun. 2024 Feb 27;15:1769. doi: 10.1038/s41467-024-46184-0 (PMC10899650; doi:10.1038/s41467-024-46184-0)

Supplement: Supplementary file 5 — Supplementary Data 1 [file 41467_2024_46184_MOESM5_ESM.zip › Supplementary Dataset/t/1.bmp]

Supplement: Supplementary file 5 — Supplementary Data 1 [file 41467_2024_46184_MOESM5_ESM.zip › Supplementary Dataset/t/10.bmp]

Supplement: Supplementary file 5 — Supplementary Data 1 [file 41467_2024_46184_MOESM5_ESM.zip › Supplementary Dataset/t/11.bmp]

Supplement: Supplementary file 5 — Supplementary Data 1 [file 41467_2024_46184_MOESM5_ESM.zip › Supplementary Dataset/t/12.bmp]

Supplement: Supplementary file 5 — Supplementary Data 1 [file 41467_2024_46184_MOESM5_ESM.zip › Supplementary Dataset/t/13.bmp]

Supplement: Supplementary file 5 — Supplementary Data 1 [file 41467_2024_46184_MOESM5_ESM.zip › Supplementary Dataset/t/14.bmp]

Supplement: Supplementary file 5 — Supplementary Data 1 [file 41467_2024_46184_MOESM5_ESM.zip › Supplementary Dataset/t/15.bmp]

Supplement: Supplementary file 5 — Supplementary Data 1 [file 41467_2024_46184_MOESM5_ESM.zip › Supplementary Dataset/t/16.bmp]

Supplement: Supplementary file 5 — Supplementary Data 1 [file 41467_2024_46184_MOESM5_ESM.zip › Supplementary Dataset/t/17.bmp]

Supplement: Supplementary file 5 — Supplementary Data 1 [file 41467_2024_46184_MOESM5_ESM.zip › Supplementary Dataset/t/18.bmp]

Supplement: Supplementary file 5 — Supplementary Data 1 [file 41467_2024_46184_MOESM5_ESM.zip › Supplementary Dataset/t/19.bmp]

Supplement: Supplementary file 5 — Supplementary Data 1 [file 41467_2024_46184_MOESM5_ESM.zip › Supplementary Dataset/t/2.bmp]

Supplement: Supplementary file 5 — Supplementary Data 1 [file 41467_2024_46184_MOESM5_ESM.zip › Supplementary Dataset/t/20.bmp]

Supplement: Supplementary file 5 — Supplementary Data 1 [file 41467_2024_46184_MOESM5_ESM.zip › Supplementary Dataset/t/21.bmp]

Supplement: Supplementary file 5 — Supplementary Data 1 [file 41467_2024_46184_MOESM5_ESM.zip › Supplementary Dataset/t/22.bmp]

Supplement: Supplementary file 5 — Supplementary Data 1 [file 41467_2024_46184_MOESM5_ESM.zip › Supplementary Dataset/t/23.bmp]

Supplement: Supplementary file 5 — Supplementary Data 1 [file 41467_2024_46184_MOESM5_ESM.zip › Supplementary Dataset/t/24.bmp]

Supplement: Supplementary file 5 — Supplementary Data 1 [file 41467_2024_46184_MOESM5_ESM.zip › Supplementary Dataset/t/25.bmp]

Supplement: Supplementary file 5 — Supplementary Data 1 [file 41467_2024_46184_MOESM5_ESM.zip › Supplementary Dataset/t/26.bmp]

Supplement: Supplementary file 5 — Supplementary Data 1 [file 41467_2024_46184_MOESM5_ESM.zip › Supplementary Dataset/t/27.bmp]

Supplement: Supplementary file 5 — Supplementary Data 1 [file 41467_2024_46184_MOESM5_ESM.zip › Supplementary Dataset/t/28.bmp]

Supplement: Supplementary file 5 — Supplementary Data 1 [file 41467_2024_46184_MOESM5_ESM.zip › Supplementary Dataset/t/29.bmp]

Supplement: Supplementary file 5 — Supplementary Data 1 [file 41467_2024_46184_MOESM5_ESM.zip › Supplementary Dataset/t/3.bmp]

Supplement: Supplementary file 5 — Supplementary Data 1 [file 41467_2024_46184_MOESM5_ESM.zip › Supplementary Dataset/t/30.bmp]

Supplement: Supplementary file 5 — Supplementary Data 1 [file 41467_2024_46184_MOESM5_ESM.zip › Supplementary Dataset/t/31.bmp]

Supplement: Supplementary file 5 — Supplementary Data 1 [file 41467_2024_46184_MOESM5_ESM.zip › Supplementary Dataset/t/32.bmp]

Supplement: Supplementary file 5 — Supplementary Data 1 [file 41467_2024_46184_MOESM5_ESM.zip › Supplementary Dataset/t/33.bmp]

Supplement: Supplementary file 5 — Supplementary Data 1 [file 41467_2024_46184_MOESM5_ESM.zip › Supplementary Dataset/t/34.bmp]

Supplement: Supplementary file 5 — Supplementary Data 1 [file 41467_2024_46184_MOESM5_ESM.zip › Supplementary Dataset/t/35.bmp]

Supplement: Supplementary file 5 — Supplementary Data 1 [file 41467_2024_46184_MOESM5_ESM.zip › Supplementary Dataset/t/36.bmp]

Supplement: Supplementary file 5 — Supplementary Data 1 [file 41467_2024_46184_MOESM5_ESM.zip › Supplementary Dataset/t/37.bmp]

Supplement: Supplementary file 5 — Supplementary Data 1 [file 41467_2024_46184_MOESM5_ESM.zip › Supplementary Dataset/t/38.bmp]

Supplement: Supplementary file 5 — Supplementary Data 1 [file 41467_2024_46184_MOESM5_ESM.zip › Supplementary Dataset/t/39.bmp]

Supplement: Supplementary file 5 — Supplementary Data 1 [file 41467_2024_46184_MOESM5_ESM.zip › Supplementary Dataset/t/4.bmp]

Supplement: Supplementary file 5 — Supplementary Data 1 [file 41467_2024_46184_MOESM5_ESM.zip › Supplementary Dataset/t/40.bmp]

Supplement: Supplementary file 5 — Supplementary Data 1 [file 41467_2024_46184_MOESM5_ESM.zip › Supplementary Dataset/t/41.bmp]

Supplement: Supplementary file 5 — Supplementary Data 1 [file 41467_2024_46184_MOESM5_ESM.zip › Supplementary Dataset/t/42.bmp]

Supplement: Supplementary file 5 — Supplementary Data 1 [file 41467_2024_46184_MOESM5_ESM.zip › Supplementary Dataset/t/43.bmp]

Supplement: Supplementary file 5 — Supplementary Data 1 [file 41467_2024_46184_MOESM5_ESM.zip › Supplementary Dataset/t/44.bmp]

Supplement: Supplementary file 5 — Supplementary Data 1 [file 41467_2024_46184_MOESM5_ESM.zip › Supplementary Dataset/t/45.bmp]

Supplement: Supplementary file 5 — Supplementary Data 1 [file 41467_2024_46184_MOESM5_ESM.zip › Supplementary Dataset/t/46.bmp]

Supplement: Supplementary file 5 — Supplementary Data 1 [file 41467_2024_46184_MOESM5_ESM.zip › Supplementary Dataset/t/47.bmp]

Supplement: Supplementary file 5 — Supplementary Data 1 [file 41467_2024_46184_MOESM5_ESM.zip › Supplementary Dataset/t/48.bmp]

Supplement: Supplementary file 5 — Supplementary Data 1 [file 41467_2024_46184_MOESM5_ESM.zip › Supplementary Dataset/t/49.bmp]

Supplement: Supplementary file 5 — Supplementary Data 1 [file 41467_2024_46184_MOESM5_ESM.zip › Supplementary Dataset/t/5.bmp]

Supplement: Supplementary file 5 — Supplementary Data 1 [file 41467_2024_46184_MOESM5_ESM.zip › Supplementary Dataset/t/50.bmp]

Supplement: Supplementary file 5 — Supplementary Data 1 [file 41467_2024_46184_MOESM5_ESM.zip › Supplementary Dataset/t/6.bmp]

Supplement: Supplementary file 5 — Supplementary Data 1 [file 41467_2024_46184_MOESM5_ESM.zip › Supplementary Dataset/t/7.bmp]

Supplement: Supplementary file 5 — Supplementary Data 1 [file 41467_2024_46184_MOESM5_ESM.zip › Supplementary Dataset/t/8.bmp]

Supplement: Supplementary file 5 — Supplementary Data 1 [file 41467_2024_46184_MOESM5_ESM.zip › Supplementary Dataset/t/9.bmp]

Supplement: Supplementary file 5 — Supplementary Data 1 [file 41467_2024_46184_MOESM5_ESM.zip › Supplementary Dataset/u/1.bmp]

Supplement: Supplementary file 5 — Supplementary Data 1 [file 41467_2024_46184_MOESM5_ESM.zip › Supplementary Dataset/u/10.bmp]

Supplement: Supplementary file 5 — Supplementary Data 1 [file 41467_2024_46184_MOESM5_ESM.zip › Supplementary Dataset/u/11.bmp]

Supplement: Supplementary file 5 — Supplementary Data 1 [file 41467_2024_46184_MOESM5_ESM.zip › Supplementary Dataset/u/12.bmp]

Supplement: Supplementary file 5 — Supplementary Data 1 [file 41467_2024_46184_MOESM5_ESM.zip › Supplementary Dataset/u/13.bmp]

Supplement: Supplementary file 5 — Supplementary Data 1 [file 41467_2024_46184_MOESM5_ESM.zip › Supplementary Dataset/u/14.bmp]

Supplement: Supplementary file 5 — Supplementary Data 1 [file 41467_2024_46184_MOESM5_ESM.zip › Supplementary Dataset/u/15.bmp]

Supplement: Supplementary file 5 — Supplementary Data 1 [file 41467_2024_46184_MOESM5_ESM.zip › Supplementary Dataset/u/16.bmp]

Supplement: Supplementary file 5 — Supplementary Data 1 [file 41467_2024_46184_MOESM5_ESM.zip › Supplementary Dataset/u/17.bmp]

Supplement: Supplementary file 5 — Supplementary Data 1 [file 41467_2024_46184_MOESM5_ESM.zip › Supplementary Dataset/u/18.bmp]

Supplement: Supplementary file 5 — Supplementary Data 1 [file 41467_2024_46184_MOESM5_ESM.zip › Supplementary Dataset/u/19.bmp]

Supplement: Supplementary file 5 — Supplementary Data 1 [file 41467_2024_46184_MOESM5_ESM.zip › Supplementary Dataset/u/2.bmp]

Supplement: Supplementary file 5 — Supplementary Data 1 [file 41467_2024_46184_MOESM5_ESM.zip › Supplementary Dataset/u/20.bmp]

Supplement: Supplementary file 5 — Supplementary Data 1 [file 41467_2024_46184_MOESM5_ESM.zip › Supplementary Dataset/u/21.bmp]

Supplement: Supplementary file 5 — Supplementary Data 1 [file 41467_2024_46184_MOESM5_ESM.zip › Supplementary Dataset/u/22.bmp]

Supplement: Supplementary file 5 — Supplementary Data 1 [file 41467_2024_46184_MOESM5_ESM.zip › Supplementary Dataset/u/23.bmp]

Supplement: Supplementary file 5 — Supplementary Data 1 [file 41467_2024_46184_MOESM5_ESM.zip › Supplementary Dataset/u/24.bmp]

Supplement: Supplementary file 5 — Supplementary Data 1 [file 41467_2024_46184_MOESM5_ESM.zip › Supplementary Dataset/u/25.bmp]

Supplement: Supplementary file 5 — Supplementary Data 1 [file 41467_2024_46184_MOESM5_ESM.zip › Supplementary Dataset/u/26.bmp]

Supplement: Supplementary file 5 — Supplementary Data 1 [file 41467_2024_46184_MOESM5_ESM.zip › Supplementary Dataset/u/27.bmp]

Supplement: Supplementary file 5 — Supplementary Data 1 [file 41467_2024_46184_MOESM5_ESM.zip › Supplementary Dataset/u/28.bmp]

Supplement: Supplementary file 5 — Supplementary Data 1 [file 41467_2024_46184_MOESM5_ESM.zip › Supplementary Dataset/u/29.bmp]

Supplement: Supplementary file 5 — Supplementary Data 1 [file 41467_2024_46184_MOESM5_ESM.zip › Supplementary Dataset/u/3.bmp]

Supplement: Supplementary file 5 — Supplementary Data 1 [file 41467_2024_46184_MOESM5_ESM.zip › Supplementary Dataset/u/30.bmp]

Supplement: Supplementary file 5 — Supplementary Data 1 [file 41467_2024_46184_MOESM5_ESM.zip › Supplementary Dataset/u/31.bmp]

Supplement: Supplementary file 5 — Supplementary Data 1 [file 41467_2024_46184_MOESM5_ESM.zip › Supplementary Dataset/u/32.bmp]

Supplement: Supplementary file 5 — Supplementary Data 1 [file 41467_2024_46184_MOESM5_ESM.zip › Supplementary Dataset/u/33.bmp]

Supplement: Supplementary file 5 — Supplementary Data 1 [file 41467_2024_46184_MOESM5_ESM.zip › Supplementary Dataset/u/34.bmp]

Supplement: Supplementary file 5 — Supplementary Data 1 [file 41467_2024_46184_MOESM5_ESM.zip › Supplementary Dataset/u/35.bmp]

Supplement: Supplementary file 5 — Supplementary Data 1 [file 41467_2024_46184_MOESM5_ESM.zip › Supplementary Dataset/u/36.bmp]

Supplement: Supplementary file 5 — Supplementary Data 1 [file 41467_2024_46184_MOESM5_ESM.zip › Supplementary Dataset/u/37.bmp]

Supplement: Supplementary file 5 — Supplementary Data 1 [file 41467_2024_46184_MOESM5_ESM.zip › Supplementary Dataset/u/38.bmp]

Supplement: Supplementary file 5 — Supplementary Data 1 [file 41467_2024_46184_MOESM5_ESM.zip › Supplementary Dataset/u/39.bmp]

Supplement: Supplementary file 5 — Supplementary Data 1 [file 41467_2024_46184_MOESM5_ESM.zip › Supplementary Dataset/u/4.bmp]

Supplement: Supplementary file 5 — Supplementary Data 1 [file 41467_2024_46184_MOESM5_ESM.zip › Supplementary Dataset/u/40.bmp]

Supplement: Supplementary file 5 — Supplementary Data 1 [file 41467_2024_46184_MOESM5_ESM.zip › Supplementary Dataset/u/41.bmp]

Supplement: Supplementary file 5 — Supplementary Data 1 [file 41467_2024_46184_MOESM5_ESM.zip › Supplementary Dataset/u/42.bmp]

Supplement: Supplementary file 5 — Supplementary Data 1 [file 41467_2024_46184_MOESM5_ESM.zip › Supplementary Dataset/u/43.bmp]

Supplement: Supplementary file 5 — Supplementary Data 1 [file 41467_2024_46184_MOESM5_ESM.zip › Supplementary Dataset/u/44.bmp]

Supplement: Supplementary file 5 — Supplementary Data 1 [file 41467_2024_46184_MOESM5_ESM.zip › Supplementary Dataset/u/45.bmp]

Supplement: Supplementary file 5 — Supplementary Data 1 [file 41467_2024_46184_MOESM5_ESM.zip › Supplementary Dataset/u/46.bmp]

Supplement: Supplementary file 5 — Supplementary Data 1 [file 41467_2024_46184_MOESM5_ESM.zip › Supplementary Dataset/u/47.bmp]

Supplement: Supplementary file 5 — Supplementary Data 1 [file 41467_2024_46184_MOESM5_ESM.zip › Supplementary Dataset/u/48.bmp]

Supplement: Supplementary file 5 — Supplementary Data 1 [file 41467_2024_46184_MOESM5_ESM.zip › Supplementary Dataset/u/49.bmp]

Supplement: Supplementary file 5 — Supplementary Data 1 [file 41467_2024_46184_MOESM5_ESM.zip › Supplementary Dataset/u/5.bmp]

Supplement: Supplementary file 5 — Supplementary Data 1 [file 41467_2024_46184_MOESM5_ESM.zip › Supplementary Dataset/u/50.bmp]

Supplement: Supplementary file 5 — Supplementary Data 1 [file 41467_2024_46184_MOESM5_ESM.zip › Supplementary Dataset/u/6.bmp]

Supplement: Supplementary file 5 — Supplementary Data 1 [file 41467_2024_46184_MOESM5_ESM.zip › Supplementary Dataset/u/7.bmp]

Supplement: Supplementary file 5 — Supplementary Data 1 [file 41467_2024_46184_MOESM5_ESM.zip › Supplementary Dataset/u/8.bmp]

Supplement: Supplementary file 5 — Supplementary Data 1 [file 41467_2024_46184_MOESM5_ESM.zip › Supplementary Dataset/u/9.bmp]
