# Supplementary figures and images for: Reconfigurable perovskite X-ray detector for intelligent imaging (part 1 of 2)
Source: Nat Commun. 2024 Feb 27;15:1769. doi: 10.1038/s41467-024-46184-0 (PMC10899650; doi:10.1038/s41467-024-46184-0)

Supplement: Supplementary file 5 — Supplementary Data 1 [file 41467_2024_46184_MOESM5_ESM.zip › Supplementary Dataset/h/1.bmp]

Supplement: Supplementary file 5 — Supplementary Data 1 [file 41467_2024_46184_MOESM5_ESM.zip › Supplementary Dataset/h/10.bmp]

Supplement: Supplementary file 5 — Supplementary Data 1 [file 41467_2024_46184_MOESM5_ESM.zip › Supplementary Dataset/h/11.bmp]

Supplement: Supplementary file 5 — Supplementary Data 1 [file 41467_2024_46184_MOESM5_ESM.zip › Supplementary Dataset/h/12.bmp]

Supplement: Supplementary file 5 — Supplementary Data 1 [file 41467_2024_46184_MOESM5_ESM.zip › Supplementary Dataset/h/13.bmp]

Supplement: Supplementary file 5 — Supplementary Data 1 [file 41467_2024_46184_MOESM5_ESM.zip › Supplementary Dataset/h/14.bmp]

Supplement: Supplementary file 5 — Supplementary Data 1 [file 41467_2024_46184_MOESM5_ESM.zip › Supplementary Dataset/h/15.bmp]

Supplement: Supplementary file 5 — Supplementary Data 1 [file 41467_2024_46184_MOESM5_ESM.zip › Supplementary Dataset/h/16.bmp]

Supplement: Supplementary file 5 — Supplementary Data 1 [file 41467_2024_46184_MOESM5_ESM.zip › Supplementary Dataset/h/17.bmp]

Supplement: Supplementary file 5 — Supplementary Data 1 [file 41467_2024_46184_MOESM5_ESM.zip › Supplementary Dataset/h/18.bmp]

Supplement: Supplementary file 5 — Supplementary Data 1 [file 41467_2024_46184_MOESM5_ESM.zip › Supplementary Dataset/h/19.bmp]

Supplement: Supplementary file 5 — Supplementary Data 1 [file 41467_2024_46184_MOESM5_ESM.zip › Supplementary Dataset/h/2.bmp]

Supplement: Supplementary file 5 — Supplementary Data 1 [file 41467_2024_46184_MOESM5_ESM.zip › Supplementary Dataset/h/20.bmp]

Supplement: Supplementary file 5 — Supplementary Data 1 [file 41467_2024_46184_MOESM5_ESM.zip › Supplementary Dataset/h/21.bmp]

Supplement: Supplementary file 5 — Supplementary Data 1 [file 41467_2024_46184_MOESM5_ESM.zip › Supplementary Dataset/h/22.bmp]

Supplement: Supplementary file 5 — Supplementary Data 1 [file 41467_2024_46184_MOESM5_ESM.zip › Supplementary Dataset/h/23.bmp]

Supplement: Supplementary file 5 — Supplementary Data 1 [file 41467_2024_46184_MOESM5_ESM.zip › Supplementary Dataset/h/24.bmp]

Supplement: Supplementary file 5 — Supplementary Data 1 [file 41467_2024_46184_MOESM5_ESM.zip › Supplementary Dataset/h/25.bmp]

Supplement: Supplementary file 5 — Supplementary Data 1 [file 41467_2024_46184_MOESM5_ESM.zip › Supplementary Dataset/h/26.bmp]

Supplement: Supplementary file 5 — Supplementary Data 1 [file 41467_2024_46184_MOESM5_ESM.zip › Supplementary Dataset/h/27.bmp]

Supplement: Supplementary file 5 — Supplementary Data 1 [file 41467_2024_46184_MOESM5_ESM.zip › Supplementary Dataset/h/28.bmp]

Supplement: Supplementary file 5 — Supplementary Data 1 [file 41467_2024_46184_MOESM5_ESM.zip › Supplementary Dataset/h/29.bmp]

Supplement: Supplementary file 5 — Supplementary Data 1 [file 41467_2024_46184_MOESM5_ESM.zip › Supplementary Dataset/h/3.bmp]

Supplement: Supplementary file 5 — Supplementary Data 1 [file 41467_2024_46184_MOESM5_ESM.zip › Supplementary Dataset/h/30.bmp]

Supplement: Supplementary file 5 — Supplementary Data 1 [file 41467_2024_46184_MOESM5_ESM.zip › Supplementary Dataset/h/31.bmp]

Supplement: Supplementary file 5 — Supplementary Data 1 [file 41467_2024_46184_MOESM5_ESM.zip › Supplementary Dataset/h/32.bmp]

Supplement: Supplementary file 5 — Supplementary Data 1 [file 41467_2024_46184_MOESM5_ESM.zip › Supplementary Dataset/h/33.bmp]

Supplement: Supplementary file 5 — Supplementary Data 1 [file 41467_2024_46184_MOESM5_ESM.zip › Supplementary Dataset/h/34.bmp]

Supplement: Supplementary file 5 — Supplementary Data 1 [file 41467_2024_46184_MOESM5_ESM.zip › Supplementary Dataset/h/35.bmp]

Supplement: Supplementary file 5 — Supplementary Data 1 [file 41467_2024_46184_MOESM5_ESM.zip › Supplementary Dataset/h/36.bmp]

Supplement: Supplementary file 5 — Supplementary Data 1 [file 41467_2024_46184_MOESM5_ESM.zip › Supplementary Dataset/h/37.bmp]

Supplement: Supplementary file 5 — Supplementary Data 1 [file 41467_2024_46184_MOESM5_ESM.zip › Supplementary Dataset/h/38.bmp]

Supplement: Supplementary file 5 — Supplementary Data 1 [file 41467_2024_46184_MOESM5_ESM.zip › Supplementary Dataset/h/39.bmp]

Supplement: Supplementary file 5 — Supplementary Data 1 [file 41467_2024_46184_MOESM5_ESM.zip › Supplementary Dataset/h/4.bmp]

Supplement: Supplementary file 5 — Supplementary Data 1 [file 41467_2024_46184_MOESM5_ESM.zip › Supplementary Dataset/h/40.bmp]

Supplement: Supplementary file 5 — Supplementary Data 1 [file 41467_2024_46184_MOESM5_ESM.zip › Supplementary Dataset/h/41.bmp]

Supplement: Supplementary file 5 — Supplementary Data 1 [file 41467_2024_46184_MOESM5_ESM.zip › Supplementary Dataset/h/42.bmp]

Supplement: Supplementary file 5 — Supplementary Data 1 [file 41467_2024_46184_MOESM5_ESM.zip › Supplementary Dataset/h/43.bmp]

Supplement: Supplementary file 5 — Supplementary Data 1 [file 41467_2024_46184_MOESM5_ESM.zip › Supplementary Dataset/h/44.bmp]

Supplement: Supplementary file 5 — Supplementary Data 1 [file 41467_2024_46184_MOESM5_ESM.zip › Supplementary Dataset/h/45.bmp]

Supplement: Supplementary file 5 — Supplementary Data 1 [file 41467_2024_46184_MOESM5_ESM.zip › Supplementary Dataset/h/46.bmp]

Supplement: Supplementary file 5 — Supplementary Data 1 [file 41467_2024_46184_MOESM5_ESM.zip › Supplementary Dataset/h/47.bmp]

Supplement: Supplementary file 5 — Supplementary Data 1 [file 41467_2024_46184_MOESM5_ESM.zip › Supplementary Dataset/h/48.bmp]

Supplement: Supplementary file 5 — Supplementary Data 1 [file 41467_2024_46184_MOESM5_ESM.zip › Supplementary Dataset/h/49.bmp]

Supplement: Supplementary file 5 — Supplementary Data 1 [file 41467_2024_46184_MOESM5_ESM.zip › Supplementary Dataset/h/5.bmp]

Supplement: Supplementary file 5 — Supplementary Data 1 [file 41467_2024_46184_MOESM5_ESM.zip › Supplementary Dataset/h/50.bmp]

Supplement: Supplementary file 5 — Supplementary Data 1 [file 41467_2024_46184_MOESM5_ESM.zip › Supplementary Dataset/h/6.bmp]

Supplement: Supplementary file 5 — Supplementary Data 1 [file 41467_2024_46184_MOESM5_ESM.zip › Supplementary Dataset/h/7.bmp]

Supplement: Supplementary file 5 — Supplementary Data 1 [file 41467_2024_46184_MOESM5_ESM.zip › Supplementary Dataset/h/8.bmp]

Supplement: Supplementary file 5 — Supplementary Data 1 [file 41467_2024_46184_MOESM5_ESM.zip › Supplementary Dataset/h/9.bmp]

Supplement: Supplementary file 5 — Supplementary Data 1 [file 41467_2024_46184_MOESM5_ESM.zip › Supplementary Dataset/s/1.bmp]

Supplement: Supplementary file 5 — Supplementary Data 1 [file 41467_2024_46184_MOESM5_ESM.zip › Supplementary Dataset/s/10.bmp]

Supplement: Supplementary file 5 — Supplementary Data 1 [file 41467_2024_46184_MOESM5_ESM.zip › Supplementary Dataset/s/11.bmp]

Supplement: Supplementary file 5 — Supplementary Data 1 [file 41467_2024_46184_MOESM5_ESM.zip › Supplementary Dataset/s/12.bmp]

Supplement: Supplementary file 5 — Supplementary Data 1 [file 41467_2024_46184_MOESM5_ESM.zip › Supplementary Dataset/s/13.bmp]

Supplement: Supplementary file 5 — Supplementary Data 1 [file 41467_2024_46184_MOESM5_ESM.zip › Supplementary Dataset/s/14.bmp]

Supplement: Supplementary file 5 — Supplementary Data 1 [file 41467_2024_46184_MOESM5_ESM.zip › Supplementary Dataset/s/15.bmp]

Supplement: Supplementary file 5 — Supplementary Data 1 [file 41467_2024_46184_MOESM5_ESM.zip › Supplementary Dataset/s/16.bmp]

Supplement: Supplementary file 5 — Supplementary Data 1 [file 41467_2024_46184_MOESM5_ESM.zip › Supplementary Dataset/s/17.bmp]

Supplement: Supplementary file 5 — Supplementary Data 1 [file 41467_2024_46184_MOESM5_ESM.zip › Supplementary Dataset/s/18.bmp]

Supplement: Supplementary file 5 — Supplementary Data 1 [file 41467_2024_46184_MOESM5_ESM.zip › Supplementary Dataset/s/19.bmp]

Supplement: Supplementary file 5 — Supplementary Data 1 [file 41467_2024_46184_MOESM5_ESM.zip › Supplementary Dataset/s/2.bmp]

Supplement: Supplementary file 5 — Supplementary Data 1 [file 41467_2024_46184_MOESM5_ESM.zip › Supplementary Dataset/s/20.bmp]

Supplement: Supplementary file 5 — Supplementary Data 1 [file 41467_2024_46184_MOESM5_ESM.zip › Supplementary Dataset/s/21.bmp]

Supplement: Supplementary file 5 — Supplementary Data 1 [file 41467_2024_46184_MOESM5_ESM.zip › Supplementary Dataset/s/22.bmp]

Supplement: Supplementary file 5 — Supplementary Data 1 [file 41467_2024_46184_MOESM5_ESM.zip › Supplementary Dataset/s/23.bmp]

Supplement: Supplementary file 5 — Supplementary Data 1 [file 41467_2024_46184_MOESM5_ESM.zip › Supplementary Dataset/s/24.bmp]

Supplement: Supplementary file 5 — Supplementary Data 1 [file 41467_2024_46184_MOESM5_ESM.zip › Supplementary Dataset/s/25.bmp]

Supplement: Supplementary file 5 — Supplementary Data 1 [file 41467_2024_46184_MOESM5_ESM.zip › Supplementary Dataset/s/26.bmp]

Supplement: Supplementary file 5 — Supplementary Data 1 [file 41467_2024_46184_MOESM5_ESM.zip › Supplementary Dataset/s/27.bmp]

Supplement: Supplementary file 5 — Supplementary Data 1 [file 41467_2024_46184_MOESM5_ESM.zip › Supplementary Dataset/s/28.bmp]

Supplement: Supplementary file 5 — Supplementary Data 1 [file 41467_2024_46184_MOESM5_ESM.zip › Supplementary Dataset/s/29.bmp]

Supplement: Supplementary file 5 — Supplementary Data 1 [file 41467_2024_46184_MOESM5_ESM.zip › Supplementary Dataset/s/3.bmp]

Supplement: Supplementary file 5 — Supplementary Data 1 [file 41467_2024_46184_MOESM5_ESM.zip › Supplementary Dataset/s/30.bmp]

Supplement: Supplementary file 5 — Supplementary Data 1 [file 41467_2024_46184_MOESM5_ESM.zip › Supplementary Dataset/s/31.bmp]

Supplement: Supplementary file 5 — Supplementary Data 1 [file 41467_2024_46184_MOESM5_ESM.zip › Supplementary Dataset/s/32.bmp]

Supplement: Supplementary file 5 — Supplementary Data 1 [file 41467_2024_46184_MOESM5_ESM.zip › Supplementary Dataset/s/33.bmp]

Supplement: Supplementary file 5 — Supplementary Data 1 [file 41467_2024_46184_MOESM5_ESM.zip › Supplementary Dataset/s/34.bmp]

Supplement: Supplementary file 5 — Supplementary Data 1 [file 41467_2024_46184_MOESM5_ESM.zip › Supplementary Dataset/s/35.bmp]

Supplement: Supplementary file 5 — Supplementary Data 1 [file 41467_2024_46184_MOESM5_ESM.zip › Supplementary Dataset/s/36.bmp]

Supplement: Supplementary file 5 — Supplementary Data 1 [file 41467_2024_46184_MOESM5_ESM.zip › Supplementary Dataset/s/37.bmp]

Supplement: Supplementary file 5 — Supplementary Data 1 [file 41467_2024_46184_MOESM5_ESM.zip › Supplementary Dataset/s/38.bmp]

Supplement: Supplementary file 5 — Supplementary Data 1 [file 41467_2024_46184_MOESM5_ESM.zip › Supplementary Dataset/s/39.bmp]

Supplement: Supplementary file 5 — Supplementary Data 1 [file 41467_2024_46184_MOESM5_ESM.zip › Supplementary Dataset/s/4.bmp]

Supplement: Supplementary file 5 — Supplementary Data 1 [file 41467_2024_46184_MOESM5_ESM.zip › Supplementary Dataset/s/40.bmp]

Supplement: Supplementary file 5 — Supplementary Data 1 [file 41467_2024_46184_MOESM5_ESM.zip › Supplementary Dataset/s/41.bmp]

Supplement: Supplementary file 5 — Supplementary Data 1 [file 41467_2024_46184_MOESM5_ESM.zip › Supplementary Dataset/s/42.bmp]

Supplement: Supplementary file 5 — Supplementary Data 1 [file 41467_2024_46184_MOESM5_ESM.zip › Supplementary Dataset/s/43.bmp]

Supplement: Supplementary file 5 — Supplementary Data 1 [file 41467_2024_46184_MOESM5_ESM.zip › Supplementary Dataset/s/44.bmp]

Supplement: Supplementary file 5 — Supplementary Data 1 [file 41467_2024_46184_MOESM5_ESM.zip › Supplementary Dataset/s/45.bmp]

Supplement: Supplementary file 5 — Supplementary Data 1 [file 41467_2024_46184_MOESM5_ESM.zip › Supplementary Dataset/s/46.bmp]

Supplement: Supplementary file 5 — Supplementary Data 1 [file 41467_2024_46184_MOESM5_ESM.zip › Supplementary Dataset/s/47.bmp]

Supplement: Supplementary file 5 — Supplementary Data 1 [file 41467_2024_46184_MOESM5_ESM.zip › Supplementary Dataset/s/48.bmp]

Supplement: Supplementary file 5 — Supplementary Data 1 [file 41467_2024_46184_MOESM5_ESM.zip › Supplementary Dataset/s/49.bmp]

Supplement: Supplementary file 5 — Supplementary Data 1 [file 41467_2024_46184_MOESM5_ESM.zip › Supplementary Dataset/s/5.bmp]

Supplement: Supplementary file 5 — Supplementary Data 1 [file 41467_2024_46184_MOESM5_ESM.zip › Supplementary Dataset/s/50.bmp]

Supplement: Supplementary file 5 — Supplementary Data 1 [file 41467_2024_46184_MOESM5_ESM.zip › Supplementary Dataset/s/6.bmp]

Supplement: Supplementary file 5 — Supplementary Data 1 [file 41467_2024_46184_MOESM5_ESM.zip › Supplementary Dataset/s/7.bmp]

Supplement: Supplementary file 5 — Supplementary Data 1 [file 41467_2024_46184_MOESM5_ESM.zip › Supplementary Dataset/s/8.bmp]

Supplement: Supplementary file 5 — Supplementary Data 1 [file 41467_2024_46184_MOESM5_ESM.zip › Supplementary Dataset/s/9.bmp]
